# Outbreak of gill myxoboliosis causing mass mortality in *Labeo catla*: pathological effects and immune modulation in host

**DOI:** 10.1186/s12866-026-04759-5

**Published:** 2026-01-27

**Authors:** Vikash Kumar, Basanta Kumar Das, Souvik Dhar, Anupam Adhikari, Payoja Mohanty, Asim Kumar Jana

**Affiliations:** Aquatic Environmental Biotechnology and Nanotechnology (AEBN) Division, ICAR-Central Inland Fisheries Research Institute (CIFRI), Barrackpore, 700120 India

**Keywords:** *Myxobolus bengalensis*, *Labeo catla*, Microscopy, Mass mortality, Immune modulation

## Abstract

*Myxobolus bengalensis* is a significant fish parasite responsible for disease and economic losses in aquaculture; however, information on its pathogenicity and impact on fish health remains limited. This study aimed to identify the morphological and molecular features of *Myxobolus bengalensis* and investigate the immunological changes in infected *Labeo catla*. Symptomatic fish showed signs of disease, including extreme lethargy, a slender body with a large head (growth retardation), and pale gills with the presence of whitish cysts in the gill lamellae. The Myxozoan parasite was isolated from infected *L. catla* tissue samples and preliminarily identified as *M. bengalensis* based on morphology, 18S rRNA PCR sequencing, and phylogenetic analysis. Tissue specimens were collected and underwent examination of immune-related gene expression (in the liver and kidney). The *M. bengalensis* spores isolated from the gills were ellipsoidal, with ovoid polar capsules. Sequencing and phylogenetic analysis of the 18S rRNA region revealed a ~ 1700 bp fragment, confirming the parasite's identity and placing it within the same clade as other *M. bengalensis* isolates. The transcription analysis of genes associated with inflammation (*TNF-α*, *IL-1β*, *iNOS*), immune activation (*TLR 4*, *C3*, *MYD88*, *NOD 1*), and both innate and adaptive immune responses (*IFN‐γ*, *Hsp70*, *Mx*, *IgM*) further highlights that *M. bengalensis* modulates the expression of genes in the liver and kidney tissue samples of infected *L. catla*. There is limited data available on the host–pathogen response during *M. bengalensis* infection; thus, this study provides key insights into the morphology, pathogenicity, and immunological impacts of *M. bengalensis* in *L. catla*.

## Introduction

In aquaculture, production losses are significant due to disease outbreaks, which negatively impact food insecurity and harm farmers’ livelihoods [16, 20]. According to the FAO report, the consequences of diseases particularly affect poor nations, causing approximately half of all productivity losses, which represent 90% of all types of aquaculture [13]. Disease-related income losses may amount to $6 billion each year [45]. Various diseases caused by microbial pathogens, including parasites, are the major bottleneck to the development and sustainability of aquaculture. Fish parasites are one of the pathogen groups that pose significant concern to the aquaculture industry, affecting fish growth and reducing productivity, as well as rendering products unsellable [8, 40, 43].

Recently, multiple reports have highlighted the emergence of new parasites, demonstrating how a diverse and extensive group of these organisms establishes strong, obligatory interactions with host species [41, 52]. Myxozoa (class Myxosporea) comprises over 2,500 species of microscopic, obligate parasitic cnidarians [54]. The few documented life cycles feature alternating spore stages that develop within obligate vertebrate hosts (primarily fish) and invertebrate hosts like annelids. Myxospores are produced in the fish host and released into the water to infect annelids, where actinospores then develop and are later released to infect new fish hosts [3, 9]. Although most myxozoan parasites infect their fish hosts without causing disease signs, there are pathogenic species that cause devastating conditions in both natural and farmed fish populations, resulting in severe ecological and economic impacts [4–6]. Several myxozoans have been reported to infect fish and cause growth retardation and mortality in aquaculture farms. For instance, the pathogenic myxozoan *Myxobolus bejeranoi* is reported to cause mortality in commercial hybrid tilapia in farm ponds in the Beit Shean Valley, located in the Syrian-African Rift Valley in northern Israel [36, 37]. The most common and abundant species among the myxozoans was *Myxobolus bengalensis* [7]. The spores of *M. bengalensis* are predominantly pyriform, characterized by a pear-shaped morphology, and are equipped with two polar capsules, a shell valve, and a sporoplasm. These spores are generally histozoic, signifying their capability to infect tissue [11, 12]. Although there are a few reports highlighting that this myxozoan parasite infects several fish species, exhibiting clinical signs of slow growth and mortality, very little information is available on parasite-mediated host responses.

In this study, a holistic characterization was undertaken to evaluate the infection status of parasitic pathogens in the freshwater fish farm of ICAR-CIFRI, India. The infections lead to severe growth retardation and mortality in cultured catla (*Labeo catla*) species. The objectives for this study were to (1) identify the causative agent responsible for growth retardation and mortality of *L. catla*; (2) characterize the causative agent through conventional, microscopic, and molecular methods; and (3) investigate the host response during infection. Potential pathogens *Myxobolus* was isolated from catla fingerlings and identified as *M. bengalensis*. Given that parasitic infections induce visible growth and health abnormalities, our goal is to better understand the factors that could contribute to their occurrence and characterize the underlying host response related to infection and fish health.

## Materials and methods

### Outbreak description

In early July 2023, we investigated a case of severe mortality, > 80% (420 nos. out of 500), in *Labeo catla* fingerlings of 120.4 ± 2.9 mm length and 21.58 ± 2.4 g weight kept at a culture facility of ICAR-CIFRI, Barrackpore, India. Based on the available records, the water quality parameters were maintained in accordance with standard guidelines. During our visit to the culture site, we observed disease symptoms, including extreme lethargy, a slender body with a relatively large head (indicating growth retardation), and pale gills with whitish cysts in the gill lamellae. Suspecting a microbial infection, moribund symptomatic fingerlings of *L. catla* showing clinical signs were transported to the laboratory, necropsied, and subjected to further microbiological tests. Fresh smears of skin and gills were examined under a microscope in the lab to check for parasites. The fish were then euthanized by an overdose of MS222 (150 mg/L) (Sigma-Aldrich), and post-mortem exams were carried out to document various clinical signs in the internal organs. For molecular studies, kidney and liver tissue samples from symptomatic and asymptomatic *L. catla* were collected in RNA later. All experiments were performed according to the animal utilization protocol approved by the Institutional Animal Ethics Committee, ICAR-CIFRI, Kolkata, India (CIFRI-IAEC/17/2023–24) for the experimental setup. All procedures were carried out with maximal effort to minimize fish suffering during the investigation.

### Parasite isolation and light microscopic identification

The whitish cysts that developed in the gills and fins were carefully collected. The external surface is gently rinsed with clean water or physiological saline to remove debris. Using sterile forceps and fine scissors, the gill covers are carefully opened, and the gill filaments are examined under a dissecting microscope or a hand lens. Visible whitish cysts are gently detached from the gill lamellae without rupturing them. For fin cysts, the affected fin rays or membrane containing the cysts are excised using sterile scissors. Some cysts were preserved in 70% alcohol, while others were prepared for light microscopy. The cysts were placed on clean glass slides with a drop of 0.9% NaCl solution, covered with coverslips, ruptured, and examined for the presence of myxosporean spores. Some spores were treated with 8% KOH to extrude the polar filament. The spores were fixed in absolute methanol for two to eight minutes and stained with Giemsa solution for approximately 25–30 min to produce a permanent specimen. The slides were then rinsed in tap water, dried, and mounted with DPX. The sporogonic plasmodia were carefully isolated using sterile needles and placed on clean slides with a drop of distilled water. The spores were covered with coverslips and sealed with DPX mount and observed under oil immersion (100 ×) using a light microscope (Leica DM 3000 LED). Identification of *Myxobolus bengalensis* was conducted according to descriptions provided by earlier researchers [10, 15, 16, 48]. It is identified primarily by the characteristic morphology of its spores and its tissue specificity in freshwater fishes. The spores are oval to pyriform in shape, medium-sized, and composed of two smooth, symmetrical shell valves joined by a distinct longitudinal suture line. Each spore contains two equal or nearly equal pyriform polar capsules positioned anteriorly, with tightly coiled polar filaments, and a binucleate sporoplasm located posterior to the polar capsules. The parasite forms whitish plasmodia (cysts) in specific tissues of the host, commonly the gills or muscles, which may be visible in heavy infections.

### Scanning electron microscopy

The myxozoans were fixed in a 2.5% glutaraldehyde solution for 2 h at 4 °C, then dehydrated in ethanol and air-dried. The sample was then coated with metallic platinum in an IB-2 ion counter and examined with a ZEISS Scanning Electron Microscope (ZEISS, Germany) at an accelerating voltage of 15.00 kV and a 10.00 KX magnification. The plasmodia attached to the fin tissue of the host fish were examined and fixed in 2.5% glutaraldehyde solution for two hours at 4 °C, followed by dehydration with ethanol and washing with absolute acetone and an amyl acetate mixture in 3:1, 2:2, and 1:3 ratios, respectively, and finally with 100% amyl acetate. The tissues were then dried at the critical point using CO2 in an HCP:2 Critical Point Dryer (Hitachi), coated with metallic platinum in Q150T ES (Quorum, UK), and examined under a Scanning Electron Microscope at accelerating voltages of 15 and 20 kV.

### 18S rRNA gene sequencing and phylogenetic analysis

The application of molecular techniques enhances species-level identification and confirms whether single or mixed infections prevail. This approach also contributes to a deeper understanding of the evolutionary relationships among closely related species (Sripalwit et al. 2015; Patarwut et al. 2020; Pitaksakulrat et al. 2022). The whitish cysts containing myxozoans were individually isolated and transferred to a 2 ml microcentrifuge tube for DNA isolation. DNA from myxozoans was prepared by lysing the cells in 1% sodium dodecyl sulfate, 0.5 M EDTA, and 10 mM Tris (pH 9.5) for 20 min at 65 °C, followed by incubation in 0.5 mg/ml pronase in a mixture of 0.6% sodium dodecyl sulfate, 0.5 M EDTA, and 10 mM Tris (pH 9.5) for 18 h at 56 °C. DNA was isolated using the phenol–chloroform extraction method, precipitated with ethanol, and resuspended in Tris-1 mM EDTA 10 mM (pH 8.0).

PCR amplification of parasitic nucleotides was performed using universal primers (Table 1) that target the highly conserved 18S rRNA gene regions [39]. The quality of the extracted DNA was checked on a 1% agarose gel and quantified using a NanoDrop (Eppendorf, Germany). The 18S rRNA gene was amplified using the GeneAmp PCR System 9700 thermal cycler (Applied Biosystems, Foster City, CA). A total of 50 μl of the PCR reaction mixture consists of 5 μl of 10 × PCR buffer, 1 μl of 10 mM dNTP, 1 μl of 50 mM MgCl_2_, 1 μl of 10 pmol of each primer, 1 U of Taq DNA polymerase, and 100 ng of isolated genomic DNA. The PCR conditions include 5 min at 94 °C for initial denaturation, 35 cycles of 94 °C for 30 s (denaturation), 54 °C for 30 s (annealing), and 72 °C for 60 s (extension), followed by 5 min at 72 °C for final extension. Amplified products were visualized on a 1.8% agarose gel [23, 26]. The amplified gene was sequenced in both forward and reverse directions using an ABI 373xl capillary sequencer (Applied Biosystems, Foster City, CA). A contig was prepared by aligning the forward and reverse sequences using DNA Baser 7.0.0. The sequence was submitted to GenBank, and a phylogenetic tree was constructed using the Neighbor-Joining method in MEGA X.

**Table 1:** List of primers used in the study

**Target gene**	**Sequence (**5ʹ−3ʹ**)**	**Product size (**~ **bp)**	**Annealing temperature**	**References**
18S rRNA	F: ACC TGG TTG ATC CTG CCA G R: CTT CCG CAG GTT CAC CTA CGG	1700	51 °C	[39]
Hsp70	F: CATGTGAGCGAGCCAAGAGA R: TTCAAAGCGAGCTCTGGTGA	112	60 °C	[50]
C3	F: TTGGCTGGACTGTGAAACCA R: AGGTGTATGATCCCACTTCCC	130	55 °C	[5]
IFN-γ	F: AAGGGTTCCTGCTCTTGTCA R: GCCATTTTTCACCTCGACTG	210	54 °C	[14]
TLR4	F: ATGATGGAGCGCAATGCCAA R: ATGTTACTCAAAGGGTCTCTGCTCC	140	55 °C	[14]
IL-1β	F: ACCCCACAAAACATCGGCCAACC R: TCTTCTCCATTTCCACCCTCTC	156	60 °C	[14]
IgM	F: TCATGATGATAAAGATGTAATGCGT R: TAATTTCCCGCCTTGTGCTC	165	55 °C	[5]
iNOS	F: GCTGCGATTCGGTCAAGTG R: CAGGAAAAGAAGGTCTGGCAGAT	130	55 °C	[5]
Mx	F: GTCCAGTACCACATGCTGGACC R: TTTGCCAGCACTCCTCAGGCGT	165	55 °C	[42]
NOD	F: GTTGGTGGGAAATACCTTGCC R: TGCTTTCGCCAGACTTCTTCC	217	56 °C	[14]
MyD88	F: CTTCCAGTTTGTGCATGAGA R: CCATCCTCTTGCACCTTTTT	146	51 °C	[14]
TNF-α	F: GCCAAGGCAGCCATCCATTTAG R: AAGTAGAGGCCGATTTGTGGGAT	155	51 °C	[14]
β-actin	F: ACCCACACTGTGCCCATCTACG R: ATTTCCCTCTCGGCTGTGGTGG	146	60 °C	[14]

### RNA isolation and cDNA synthesis

RNA was isolated from liver and kidney tissues of symptomatic and asymptomatic fish samples using the following protocol [23], with slight modifications. Briefly, 100 mg (micrograms) of each tissue was weighed and homogenized aseptically for 35–45 s in RT with 1000 L of chilled Tri-Reagent (Sigma, USA). The homogenate was incubated for 6 min at 18 °C. Later, chilled chloroform (Product No. 366927, Sigma-Aldrich, USA) was added to the homogenate, mixed vigorously for 18 min at 22 °C, and centrifuged (Model No. Z207, Hermble, Germany) for 12 min at 12,000 rpm. The supernatant aqueous layer was transferred to a sterile 1.5 mL Eppendorf tube and 500 µL of 2-propanol (Product No. I9516, Sigma-Aldrich, USA) was added. The mixture was kept at −20 °C overnight. The next day, the mixture was centrifuged for 15 min at 15,000 rpm. Afterward, the pellet was obtained and washed with 75% ethanol, centrifuged at 7000 rpm for 9 min, and air-dried to remove the excess ethanol. 50 µl of sterile DEPC-treated water were used to dissolve the RNA pellets. Then, the RNA was treated with DNAse I (Thermo Scientific, India) to eliminate genomic DNA contamination. A spectrophotometer was used to measure the RNA content (in ng/µL) and quality at 260/280 nm. The RNA's integrity was examined with a 2% agarose gel.

Interestingly, the reverse transcription method was used to prepare cDNA (complementary DNA) with the RevertAid™ H Minus First Strand Synthesis Kit (Catalog number: K1631, Thermo Scientific, India). To put it briefly, 1 µL of random hexamer primer solution and 1 µg of total RNA were combined. Next, 8 µL of reaction mixture was added, which included 20 units of ribonuclease inhibitor, 2 µL of 0.01 mol-1 dNTP mix, 200 units of RevertAid™ H minus M-MuLV reverse transcriptase, and 5 × reaction buffer 4 µL (0.25 mol-1 Tris–HCl, pH 8.3, 0.25 mol-1 MgCl_2_, 0.05 mol-1 DTT). The reaction was stopped after 5 min of heating at 70 °C and then cooled to 4 °C. Before use, the complementary deoxyribonucleic acid (cDNA) samples were stored at −20 °C and then subjected to PCR analysis.

### Quantitative real-time PCR

The expression of NOD-like receptors (*NOD*), Myeloid differentiation primary response 88 (*MYD88*), complement factor 3 (*C3*), Inducible nitric oxide synthase (*iNOS*), heat shock protein 70 (*Hsp70*), Interferon‐gamma or type II interferon (*IFN‐γ*), myxovirus resistance (*Mx*), Tumour necrosis factor α (*TNF-α*), Immunoglobulin (*IgM*), Toll-like receptor (*TLR 4*), Interleukin-1β (*IL-1β*), and *β-actin* (housekeeping gene) were measured by RT-qPCR with a pair of specific primers using StepOnePlus Real-time PCR systems (Applied Biosystems) (N. [19, 25]) (Table 1).

Twenty millilitres were the total volume used for the amplification, which included 0.5 millilitres of each particular primer, 10 millilitres of 2 × Maxima SYBR Green/ROX qPCR Master Mix (Thermo Fisher Scientific), one millilitre of cDNA (50 ng), and eight millilitres of nuclease-free water. A four-step RT-qPCR amplification protocol was used for the target and reference genes. First, master mixes were prepared for each biological replicate of the sample in triplicate. Next, 40 cycles of amplification and quantification were carried out, with 15 s at 95 °C, 30 s at 60 °C, and 30 s at 72 °C. Finally, a melting curve (55–95 °C) was used, with a heating rate of 0.10 °C/s and a continuous fluorescence measurement. Finally, cooling (4 °C) was applied. A negative control reaction was included for each primer set by omitting the template cDNA. The target genes' expression levels were examined using the comparative CT approach (2^^∆∆Ct^ method), as described by Livak and Schmittgen [30]. The Pfaffl relative standard curve approach was used to verify [44]. A t-test was performed on the log-transformed 2^^∆∆Ct^ value, and *P* values less than 0.05 were regarded as statistically significant.

### Statistical analysis

The data were arcsin-transformed to ensure homoscedasticity and normality, as necessary. After that, it was subjected to Duncan's multiple range test and a one-way analysis of variance (ANOVA) using the statistical software program for the social sciences, version 24.0. Results for the gene expressions were represented as fold changes relative to the internal control gene (β-actin). The expression level in the control was regarded as 1.0, and thereby, the expression ratio of the treatments was expressed in relation to the control. Analysis of significant differences in expression levels between the control and treatment groups was performed using single-tailed Student's t-tests on log-transformed data. The significance level was set at *P* ≤ 0.05.

## Results

### Isolation and characterization of Myxozoan parasite induces mass mortalities

**Fig. 1:**
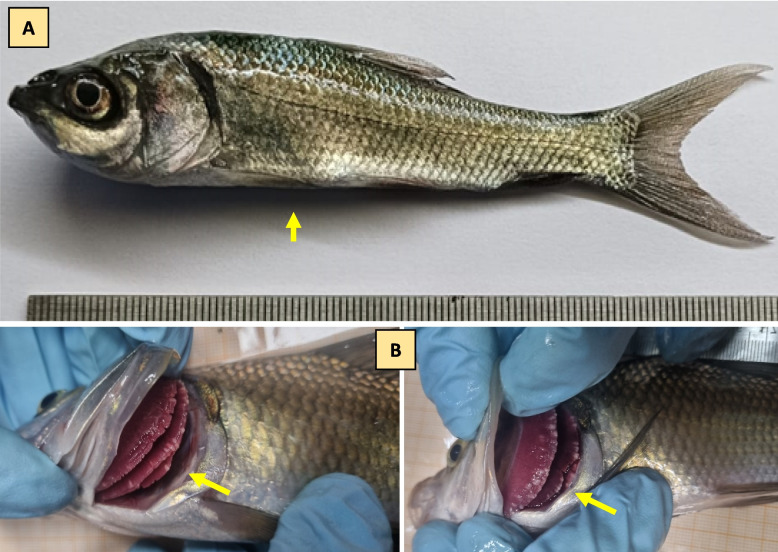
Infected moribund bata (*Labeo catla*) samples showing clinical signs (yellow arrowhead) were collected from the culture facility. **A** The infected fish exhibits growth retardation, characterized by a slender body and a large head. **B** Pale color gill with the presence of whitish cysts in the gill lamellae

**Fig. 2:**
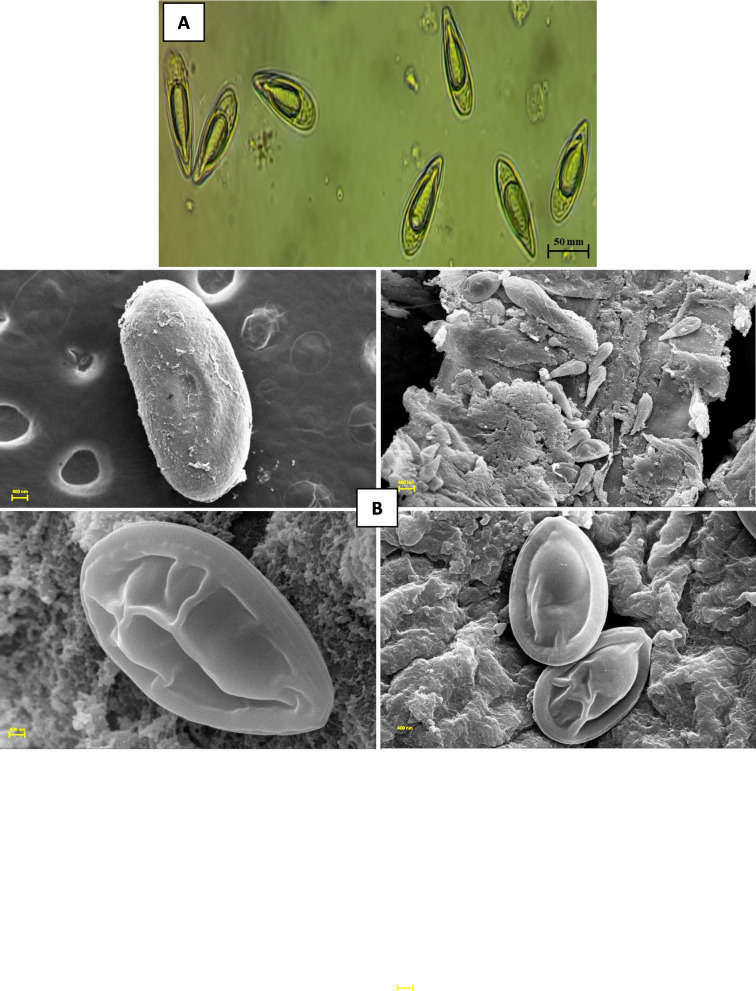
Examination of isolated *Myxobolus bengalensis* under (**A**) light microscopy analysis revealed the morphology of isolated parasite spores, ellipsoidal in frontal view, lemon-shaped in lateral view, and the presence of polar capsules, possibly indicating the presence of the *Myxobolus* genus. **B** Scanning electron microscopy (SEM) analysis provided a further overview of the morphology of parasite spores and cysts

During the disease outbreak, we analyzed the physicochemical parameters of the water and found that pH, dissolved oxygen (DO), water temperature, conductivity, salinity, total nitrogen, NH_4_-N, NO_3_-N, and total phosphorus remained within the optimal range for fish culture. Initial observations highlighted that fish were extremely lethargic, had a slender body with a large head (growth retardation), and pale gill color, with whitish cysts in the gill lamellae, suggesting possible microbial pathogen involvement (Fig. 1). The light and electron microscopy study revealed that *M. bengalensis* comprises pear-shaped myxospores with longer, equal polar capsules and a spherical iodinophilous vacuole [17]. The investigation reveals the presence of a Myxozoan parasite linked to the disease outbreak in *L. catla* (Fig. 2).

**Fig. 3:**
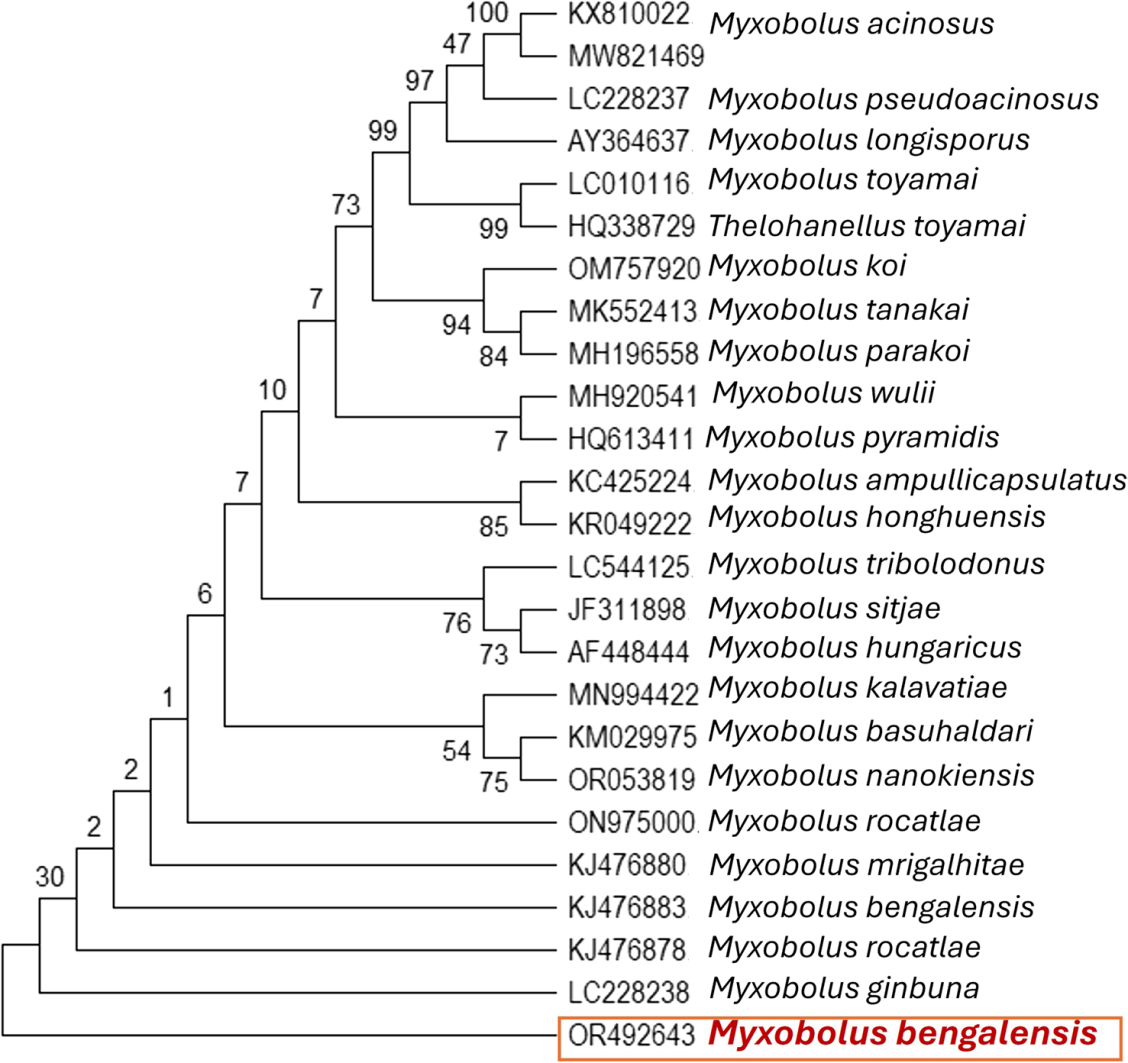
Phylogenetic tree analysis of fungal isolates based on ITS1 and ITS4 nucleotide sequences following the maximum composite likelihood method by the MEGA11 software. The numbers next to the branches indicate percentage values for 1000 bootstrap replicates. The isolated parasite was identified as *Myxobolus bengalensis*

The isolated Myxozoan parasite underwent further examination with 18S rRNA gene sequencing and phylogenetic analysis. The sequenced 18S rRNA gene from the isolated parasite was submitted to GenBank and received accession number OR492643. BLAST analysis against a non-redundant database revealed a similarity of greater than 98% with *Myxobolus bengalensis* (GenBank Accession Numbers: MK412934 and KJ476883). Subsequently, a phylogenetic tree was created using the sequence from NCBI (Fig. 3). Furthermore, we tested the extracted DNAs for the presence of additional major fish bacterial (*Aeromonas hydrophila*, *A. veronii*, *Pseudomonas aeruginosa*, *Edwardsiella tarda*) and viral pathogens (including Spring viremia of carp and Viral hemorrhagic septicemia). Remarkably, all catla samples tested negative for these major microbial pathogens.

### Effect of Myxozoan parasite on catla health and immunity

**Fig. 4:**
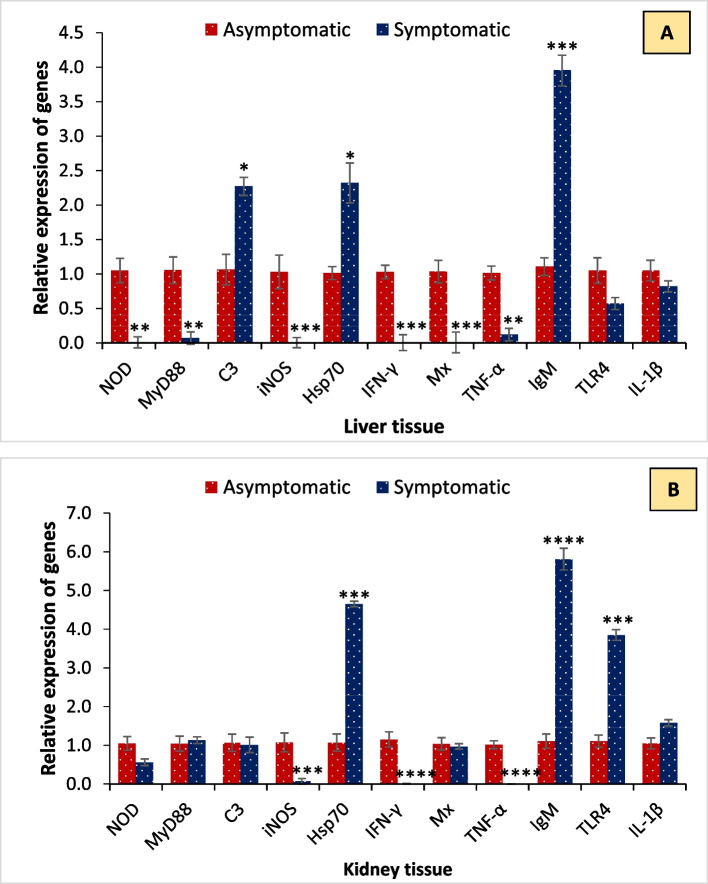
Fold change in gene expression of catla tissue samples in asymptomatic and symptomatic groups. Expression of NOD-like receptors (*NOD*), Myeloid differentiation primary response 88 (*MYD88*), complement factor 3 (*C3*), Inducible nitric oxide synthase (*iNOS*), heat shock protein 70 (*Hsp70*), Interferon‐gamma or type II interferon (*IFN‐γ*), myxovirus resistance (*Mx*), Tumour necrosis factor α (*TNF-α*), Immunoglobulin (*IgM*), Toll-like receptor (*TLR 4*) and Interleukin-1β (*IL-1β*) genes by quantitative real-time PCR. The temporal expression was analyzed from asymptomatic and symptomatic bata from (**A**) liver and (**B**) kidney tissue samples. The expression level in the asymptomatic group was regarded as 1.0, and thereby, the expression ratio of the symptomatic group was expressed as compared to the control group. The results are the mean ± SE (*n* = 3), and the vertical bars with an asterisk indicate significant differences between treatment groups (*P* < 0.05)

The temporal expression of inflammation-related (TNF-α, IL-1β, iNOS), immune activation-related (TLR4, C3, MYD88, NOD1), and innate and adaptive immune response-related genes (IFN-γ, Hsp70, Mx, IgM) exhibited differential expression profiles in symptomatic fish compared to the asymptomatic group. The qPCR analysis revealed that transcription of *C3* and *Hsp70* (> 2 folds) and *IgM* (~ 4 folds) genes was significantly upregulated in liver tissue during *M. bengalensis* infection as compared to the control (Fig. 4A). In contrast, the expression of *NOD*, *MyD88*, *iNOS*, *IFN‐γ*, *Mx*, *TNF-α*, *TLR4,* and *IL-1β* was downregulated in the symptomatic fish. Moreover, increased expression of *HSp70* and *TLR 4* (~ 4 folds) and *IgM* (~ 6 folds) genes was observed in kidney tissue as compared to control during infection (Fig. 4B). While the expression of other studies' genes was downregulated during *M. bengalensis* infection. In total, these results highlight that *M. bengalensis* infection differentially modulates immunity, which potentially leads to high mortality in catla during infection.

## Discussion

Infectious diseases lead to significant declines in populations of both wild and captive animals [23, 24]. Diseases among commercially farmed species lead to substantial economic losses annually, particularly in fish farming, where the combination of high animal densities and inadequate water quality creates favorable conditions for disease outbreaks, including those triggered by parasites [28]. One of the most prevalent issues facing aquaculture is the presence of parasitic infestations [46, 47], which can result in widespread mortality within their populations. As aquaculture systems gain importance in several nations, researchers have increasingly focused on parasitic diseases affecting cultured fish. The occurrence of myxosporean parasites in Indian fish species is relatively well studied, with 130 species listed [17, 51]. Notably, *Myxobolus* spp. infecting major carps (*Labeo* and *Cirrhinus*) are represented by > 40 species from the genera *Labeo* and *Cirrhinus *[34, 49]. Unfortunately, the majority of Indian myxosporean species have been poorly characterized; they are studied primarily through morphometric measurements and the shape of spores and polar capsules, with limited information provided about the host response, including cellular and molecular changes [6, 55]. In this study, we addressed several knowledge gaps in the host–pathogen relationship during parasitic infections in the important commercial fish species *Labeo catla*. The current study provides novel and multifaceted insights into *M. bengalensis* infection in *L. catla*. The infection leads to severe mortality > 80% in catla species. Elevated fish mortality may be attributed to the failure of respiratory function and immune response following the downregulation of key immune genes, as evidenced by gene expression analyses.

The observed clinical signs aligned with previous reports, which describe lethargy, loss of appetite, abnormal swimming patterns, skin darkening, excessive mucus secretion, respiratory distress, and stunted growth associated with myxosporean parasite infections [12, 37, 38, 53]. Similarly, macroscopic creamy white nodules containing whitish cysts of *M. bengalensis* were recorded in the gills of *L. catla*. The light and scanning electron microscopy studies were used to characterize the myxospores, polar capsules, and vacuoles. Investigation revealed that *M. bengalensis* spores isolated from the gills were ellipsoidal (~ 50.0 μm width, ~ 100.0 μm in length), with ovoid polar capsules [1, 29, 31, 32]. The presence of an indistinguishable morphological appearance in our specimens precluded the presence of multiple myxosporean species, which frequently occur as mixed infections. Molecular analysis of the 18S rRNA gene amplified from *M. bengalensis* parasites isolated from symptomatic catla revealed identical ~ 1700 bp sequences. BLAST analysis matched these sequences to the genus *Myxobolus*, and alignment confirmed the identity as *M. bengalensis*. The 18S rRNA gene sequences exhibited 98% similarity to other sequences of *M. bengalensis* (GenBank Accession Numbers: MK412934 and KJ476883). Phylogenetic reconstruction placed the *M. bengalensis* species investigated herein within the same clade as other *M. bengalensis* references with high bootstrap support. This corroborates its molecular identification and distinguishes it from related parasites.

The fish immune response is a cascade of diverse reactions that aims to eliminate the recognized foreign agent and restore homeostasis. Since microbial infections pose a persistent threat to aquatic animals, immune factors, both specific and non-specific, may offer a preventive strategy for controlling biotic and abiotic stresses in aquaculture farming systems [27, 33, 35]. However, little is known about the effect of host–pathogen responses during parasitic infections, and more extensive research is needed to determine the immunomodulation mechanisms induced by myxosporean parasites (*M. bengalensis*) in commercial candidate fish species, such as *L. catla*. The expression of inflammation-related (TNF-α, IL-1β, iNOS), immune activation-related (TLR4, C3, MYD88, NOD1), and innate and adaptive immune response-related genes (IFN-γ, Hsp70, Mx, IgM) is typically interpreted as an indication of immune stimulation or an augmented immune response. An immediate and vigorous response that triggers multiple inflammatory reactions is mediated by the classic pro-inflammatory cytokines, e.g., *TNF-α*, *IL-1β*, and *iNOS*, which play a pivotal role in the early inflammatory response. The key players in both innate and adaptive immune defense are genes associated with immune activation (*NOD, MyD88, C3, Hsp70, IFN-γ, Mx, IgM,* and *TLR4*). These genes play various roles, including opsonization, direct killing, regulation of immune responses, and mediation of inflammation [18, 19, 24–26]. In this study, *M. bengalensis* infections had a significant effect on the transcription of *C3*, *Hsp70*, *IgM*, *NOD*, *MyD88*, *iNOS*, *IFN‐γ*, *Mx*, *TNF-α*, *TLR4,* and *IL-1β* genes. Analysis revealed that *M. bengalensis* infections stimulate the expression of genes related to immune activation and homeostasis in liver and kidney tissue. *C3* and *Hsp70* (> 2 folds) and *IgM* (~ 4 folds) genes were significantly upregulated in liver tissue, while increased expression of *HSp70* and *TLR 4* (~ 4 folds) and *IgM* (~ 6 folds) genes was observed in kidney tissue. In parallel, reports suggest that myxosporean infection modulates the immune response, thereby influencing fish survival and growth [2, 48]. The present findings, in conjunction with microscopic observations, suggest that gene transcription modulation may be related to the *M. bengalensis* virulence strategy, which involves either impairing the inflammatory response or evading the host's immune response through developed mechanisms [21, 22]. In parallel, reports suggest that *Myxobolus* infection weakens the host immune system, conditions that facilitate the establishment of parasitic species in host tissue, resulting in the initiation of infection and mortality [36]

This comprehensive study, integrating clinical, microscopic, and molecular data, significantly enhances our understanding of *M. bengalensis* infections in *L. catla*. The findings elucidate clinical manifestations, tissue tropisms, cyst morphology, and gene expression profiles. The gene expression results provide valuable insights into the molecular pathways affected during infections, highlighting the importance of such studies in understanding host-parasite relationships. This multidimensional approach advances fundamental knowledge and demonstrates the benefits of integrating diverse techniques in the study of host-parasite dynamics. However, the lack of treatments to protect fish from myxosporean parasite infections in aquaculture, combined with the high mortality observed after these infections, makes it urgent to clarify additional anti-parasitic defenses, particularly against *Myxobolus* sp. in fish. Future research can build on these insights to further enhance the sustainability of aquaculture and seafood safety.

## Data Availability

The datasets generated and/or analyzed during the current study are available in the NCBI repository, accession number OR492643.
